# Pulmonary edema following transcatheter closure of atrial septal defect

**DOI:** 10.4103/0974-2069.64359

**Published:** 2010

**Authors:** Anil Kumar Singhi, Kappanayil Mahesh, R Krishna Kumar

**Affiliations:** Department of Pediatric Cardiology, Amrita Institute of Medical Sciences, Kochi, Kerala, India

**Keywords:** Atrial septal defect, pulmonary edema, catheter closure

## Abstract

Pulmonary edema after device closure of atrial septal defect (ASD) is a rare complication. We present illustrative images of a case of pulmonary edema after device closure of ASD in a 53 year old adult. Older patients undergoing ASD closure can benefit from their left atrial and left ventricular end diastolic pressures measurement before and after temporary balloon occlusion of ASD.

These images illustrate the development of pulmonary edema after catheter closure of atrial septal defect (ASD) in a 53-year-old gentleman. He presented with shortness of breath and easy fatigability since six months. He was normotensive and non-diabetic with no history of effort angina. The chest X-ray obtained before the procedure [Figure 1] showed cardiac enlargement with dilated central pulmonary arteries and plethoric lung fields. The two-dimensional transthoracic echocardiogram revealed moderate sized secundum ASD with a left to right shunt. The right atrium, right ventricle and pulmonary artery (PA) were dilated suggestive of a significant left to right shunt. His biventricular contractility was noted to be normal. The defect size was 20 mm as measured by transesophageal echocardiography. During cardiac catheterization, the PA pressure was 53/19 (mean 32) mmHg and right ventricular end diastolic pressure eight mmHg. Coronary angiography was normal. Pressure in the left atrium and left ventricle were not measured. He underwent closure of ASD with a 22 mm device (Lifetech Scientific Ltd., Shenzen, China). Balloon sizing of the defect was not performed and the effects of temporary balloon occlusion on the left atrial or pulmonary artery pressures not measured. The device position was satisfactory and there was no residual flow.

**Figure 1:**
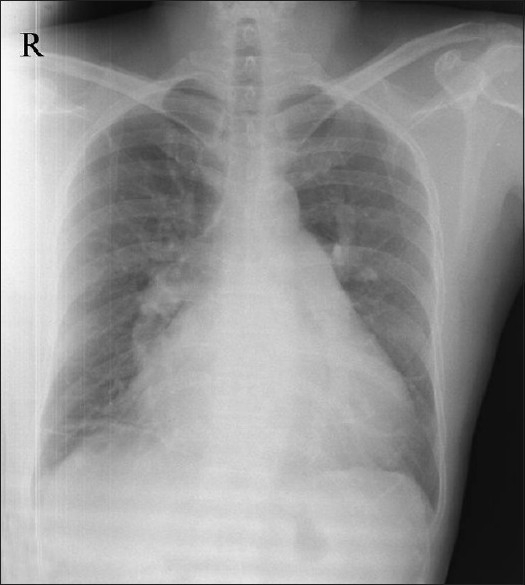
X-ray chest in postero-anterior view showing cardiomegaly and increased pulmonary blood flow, dilated right atrium and dilated pulmonary artery

Eighteen hours after device closure the patient developed breathing difficulty and orthopnea. Clinical examination showed tachypnea and basal crepitation. Skiagram of chest in postero-anterior view showed features of evolving pulmonary edema [Figure 2] which established after 24 hours after device closure [Figure 3]. The patient was shifted to intensive care unit and treated with intravenous diuretics. His symptoms improved over the next two days and he was discharged. On follow-up after three months, the right ventricular systolic pressure was measured 32 mmHg by tricuspid regurgitation jet. Following this event we have started to measure the left ventricular end diastolic pressures (LVEDP) in all patients older than 40 years before closing the ASD. We also test the effects of balloon occlusion on the LVEDP and PA pressures before closing the defect.

**Figure 2:**
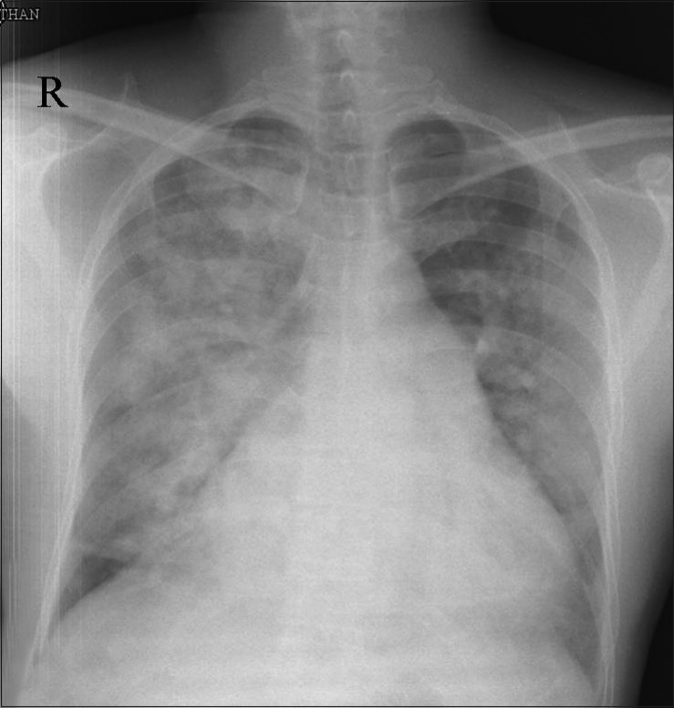
X-ray chest in postero-anterior view 18 hours after atrial septal defect device closure showing evolving pulmonary edema

**Figure 3:**
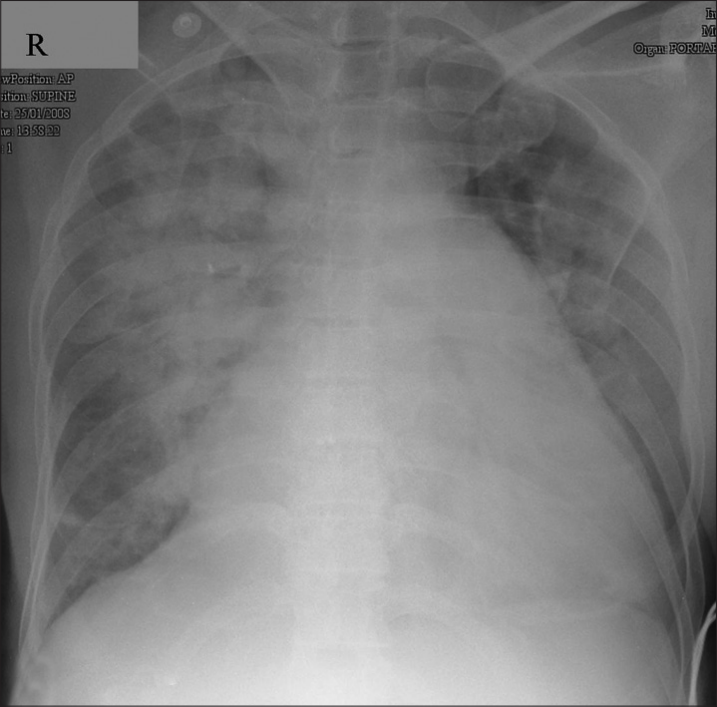
X-ray chest in postero-anterior view 24 hours after atrial septal defect device closure showing established pulmonary edema

## DISCUSSION

ASD is a common form of congenital heart disease accounting for approximately 10% of all congenital cardiac defects.[1] Transcatheter closure of secundum ASD has evolved over the past three decades, and is now a standard treatment for secundum ASD. Major complications after ASD device closure are rare. Du *et al*.[2] reported 1.6% complication rate in a cohort of 442 patients with no report of pulmonary edema. The occurrence of pulmonary edema is reported mainly in elderly patients. Ewert *et al*. reported pulmonary edema in a 78-year-old male. Our patient was relatively younger (53 years) without any coronary artery disease and normal systemic pressure.

In older patients, an impaired diastolic ventricular function due to reduced ventricular compliance is observed more frequently although this is occasionally also seen in childhood.[3] Restrictive left ventricular dysfunction in elderly may be masked by the presence of an ASD. Deterioration of left ventricular diastolic function can occur with acute hemodynamic change, following interventional closure of ASD, leading to acute lung edema.[4] The only independent risk factor to identify these patients is elevated left atrial pressure, which increases significantly during balloon test occlusion, indicating underlying restrictive left ventricular dysfunction.[4]

Older patients undergoing ASD closure in the catheterization laboratory should perhaps have their left atrial, LVEDP measured before and after temporary balloon occlusion and after defect is closed with a device.
